# Putting a stereotype to the test: The case of gender differences in multitasking costs in task-switching and dual-task situations

**DOI:** 10.1371/journal.pone.0220150

**Published:** 2019-08-14

**Authors:** Patricia Hirsch, Iring Koch, Julia Karbach

**Affiliations:** 1 Institute of Psychology, RWTH Aachen University, Aachen, Germany; 2 Department of Psychology, University of Koblenz-Landau, Landau, Germany; University College London, UNITED KINGDOM

## Abstract

According to a popular stereotype, women are better at multitasking than men, but empirical evidence for gender differences in multitasking performance is mixed. Previous work has focused on specific aspects of multitasking or has not considered gender differences in abilities contributing to multitasking performance. We therefore tested gender differences (*N* = 96, 50% female) in sequential (i.e., task switching) and concurrent (i.e., dual tasking) multitasking, while controlling for possible gender differences in working memory, processing speed, spatial abilities, and fluid intelligence. Applying two standard experimental paradigms allowed us to test multitasking abilities across five different empirical indices (i.e., performance costs) for both reaction time (RT) and accuracy measures, respectively. Multitasking resulted in substantial performance costs across all experimental conditions without a single significant gender difference in any of these ten measures, even when controlling for gender differences in underlying cognitive abilities. Thus, our results do not confirm the widespread stereotype that women are better at multitasking than men at least in the popular sequential and concurrent multitasking settings used in the present study.

## Introduction

It is a widely held belief that women outperform men in multitasking situations, possibly because of an evolutionary advantage and extensive multitasking practice resulting from managing children, household, and jobs [1, 2]. In fact, two recent studies showed that the majority of participants was convinced that gender differences in multitasking existed and at least 80% of them attributed better multitasking abilities to women than to men [1, 3].

Multitasking is a broad construct that can be operationalized and measured in numerous ways [4]. It refers to activities in which multiple tasks, each associated with a separate task set, are performed in a limited time period, leading to a temporal overlap of the cognitive processes in performing these tasks. Such temporal overlaps of cognitive processes involved in performing multiple tasks occur, for instance, in task-switching (i.e., sequential multitasking) and dual-task (i.e., concurrent multitasking) contexts (see [5] for a review). Since we act in the context of various potentially relevant task sets in task-switching and dual-task situations, successful performance in these multitasking situations requires the selection of appropriate task sets (i.e., working memory updating), the reduction of interfering influences of other task sets (i.e., inhibition), and the disengagement and engagement of task sets (i.e., shifting). Working memory updating, inhibition, and shifting have been proposed as core components of cognitive control, a multidimensional construct that is responsible for the regulation of cognitive processing in accordance with current task goals [6].

In *task-switching paradigms*, subjects perform two simple decision tasks (A and B). They perform single-task blocks that include only one of these tasks (A or B) as well as mixed-task blocks, that require task repetitions and task switches on a trial-by-trial basis (AB, BA, AA, BB) (see [5, 7] for reviews). In task switching paradigms, tasks are performed sequentially and the stimulus for a given trial is presented only after the previous task is completed. The design allows calculating two types of performance costs (mixing costs and switch costs) that are typically assessed as markers of multitasking performance.

*Mixing costs* refer to worse performance in repetition trials of mixed-task blocks (AA, BB) than in single-task blocks where subjects perform only one task (i.e., all trials are by definition repetition trials). This type of cost is a measure for working memory processes of task-set maintaining and updating required in mixed-task blocks independent of the specific shifting component [8]. *Switch costs* reflect worse performance in switch trials (AB, BA) than repetition trials (AA, BB) of mixed-task blocks and are a measure for the engagement and disengagement of task sets (i.e., shifting component) [6].

In *dual-task paradigms*, such as the psychological refractory period (PRP) paradigm, subjects perform two tasks, Task 1 and Task 2, each associated with different stimuli and responses, with temporal overlap (i.e., dual-task condition) (see [9] for a review). In this paradigm, the degree of temporally overlapping task processing is determined by the time interval between the onset of Stimulus 1 and Stimulus 2 (i.e., stimulus-onset asynchrony, SOA). Thus, in contrast to task-switching paradigms, Stimulus 2 is usually presented before a response for Stimulus 1 is made, resulting in simultaneous task processing at least for a limited time period. Moreover, there are single-task conditions in which subjects perform tasks without temporal overlap. In dual-task paradigms, dual-task costs and the PRP effect are usually assessed as markers of multitasking performance.

*Dual-task costs* are calculated as the difference between Task 2 performance in dual-task conditions and performance in single-task blocks [10]. The *PRP effect* reflects worse Task 2 performance in dual-task conditions with short SOAs than with long SOAs [9]. In contrast to the multitasking costs measured in task-switching contexts, there is so far no consensus about the underlying cognitive mechanisms (i.e., working memory updating, inhibition, and shifting) of performance costs arising in dual-task contexts. However, a recent study by Hirsch and colleagues (2018) suggests that dual-task costs reflect, like mixing costs, cognitive processes involved in maintaining and updating task sets in working memory [11]. Furthermore, this study provides first evidence indicating that the PRP effect might reflect at least partly processes related to the engagement and disengagement and/or inhibition of task sets.

Studies systematically exploring gender differences in task-switching and dual-task situations are rare and their findings are heterogeneous [12]. On the one hand, some studies reported neither gender differences in sequential nor in concurrent multitasking performance [12–14]. For instance, Paridon and Kaufmann (2010) conducted a dual-task study where subjects performed a driving simulation task (i.e., lane-change-task with lane deviation as the dependent variable) either in isolation or in temporal overlap with another task, such as, for example, dialing a number on a mobile phone, taking a tissue out of its packet, or reading directions [15]. Performance declined when the driving task was performed simultaneously with another task than when it was performed in isolation. However, these multitasking costs (i.e., dual-task vs. single-task performance) were comparable across gender groups, suggesting that women and men performed equally well in concurrent multitasking situations.

On the other hand, there is evidence for better sequential multitasking performance in women than in men [16–17]. For instance, Stoet and colleagues (2013) instructed participants to either repeat or switch between a shape discrimination task and a filling discrimination task [17]. They found higher multitasking costs (i.e., mixed-task vs. single-task performance) in men than in women, which suggest that women are better at sequential multitasking than men.

Finally, some studies observed performance costs in multitasking to be more pronounced for women than for men [18–19]. Mäntylä (2013), for instance, reported a dual-task study in which participants performed an *n*-back memory updating task while carrying out three independent monitoring tasks in which they monitored digital “clocks” (counters) with forward running digits [19]. They had to respond whenever a counter displayed a target reading that was defined by a certain rule (e.g., when the last two digits of a counter were a multiple of 11). Mäntyla (2013) found that men outperformed women in the monitoring accuracy, indicating that they were better at concurrent multitasking than women [19]. In this study, gender differences were fully mediated by gender differences in spatial abilities, suggesting that spatial ability is a central explanatory construct for the observed data pattern. More specifically, Mäntylä (2013) argued that gender differences in multitasking performance occur only when task management necessitates a very complex coordination of spatially distributed tasks. This is consistent with a more recent dual-task study reporting that men performed better than women in concurrent multitasking tapping spatial abilities [20].

Hence, some studies report no gender differences [12–15], while others report women to be better at multitasking than men [16, 17] or vice versa [18, 19]. Moreover, existing findings are difficult to compare and interpret because they either exclusively focused on sequential (e.g., task-switching, such as [17]) or concurrent multitasking (e.g., dual-tasking, such as [15]), or because they were based on very small sample sizes (e.g., gender differences in the PRP effect assessed with 10 subjects per gender group in [18] or relied on post-hoc exploratory analyses [21]).

We therefore systematically examined gender differences in multitasking performance with sufficiently large sample size (*N* = 2 x 48) to ensure the detection of at least medium-to-large gender-effects in multitasking costs in a well-powered study (i.e., 0.82 for moderate gender effects with a cohen’s *d* of 0.6 [22]). Moreover, we directly compared sequential and concurrent multitasking by applying typical and highly comparable task-switching and dual-task paradigms, including the same stimuli, tasks, and responses across paradigms, to allow for a generalization of findings across different multitasking contexts. Moreover, we considered possible gender differences in abilities supporting multitasking performance (i.e., working memory capacity, processing speed, spatial abilities, and intelligence).

## Methods

### Participants

Forty-eight women (*M* = 24.07 years; *SD* = 3.58) and 48 men (*M* = 24.99 years; *SD* = 3.59) participated in the experiment. Five additional participants (3 women and 2 men) were tested, but because of excessive error rates (> 32.2%), data were excluded from the analyses. Forty-eight participants (24 female) were recruited from the subject database of Goethe-University Frankfurt, and 48 other participants (24 female) were tested at RWTH Aachen University. All subjects received payment (8€/hour) and reported no neurological or psychiatric disorders. They had normal or corrected-to-normal vision and no hearing impairments. Women did not take hormonal contraceptives and were not pregnant at the time of the study.

All subjects provided written informed consent. All procedures performed in the present study were in accordance with the Helsinki declaration and comparable ethical standard. The study was not formally submitted to an ethics committee because no physical or psychological discomfort and harm was expected to result from the participation in this study. Moreover, we did not use invasive methods and did not test underage persons or patients.

To ensure that the gender groups were comparable regarding mental and physical health, processing speed, working memory capacity, intelligence, and spatial abilities, and that possible gender differences in multitasking performance were not due to gender differences in these variables, all subjects completed a demographic questionnaire and a cognitive screening. Concerning mental health, prior studies provided evidence that depression and negative mood modify cognitive control processes [23, 24]. With regard to the cognitive screening, previous studies reported that working memory capacity, intelligence, and spatial abilities predicted multitasking performance [19, 25].

Comparisons (two-tailed *t*-tests) showed no gender difference in terms of age between women and men (24.07 years for women vs. 24.99 years for men, *t*(94) = -1.26, *p* = .595, *d* = 0.26), physical and mental health (self-rated on one item with a Likert scale ranging from *0* meaning very well to *4* indicating very bad; physical: 0.79 for women vs. 0.73 for men, *t*(94) = 0.49, *p* = .628, *d* = -0.10; mental: 0.67 for women vs. 0.79 for men, *t*(94) = -1.01, *p* = .315, *d* = 0.21), working memory capacity (percentile rank reflecting the difference between proportion of hits and false alarms on an adaptive auditory *n*-back task [26]; 0.23 for women vs. 0.23 for men, *t*(94) = -0.09, *p* = .933, *d* = 0.02), and intelligence (*n* correct on the matrix test of the German version of the Wechsler Intelligence Scale for Adults [27]; 20.98 for women vs. 21.08 for men, *t*(94) = -0.20, *p* = .839, *d* = 0.04). However, women showed a faster processing speed compared to men (*n* correct on the Digit-Symbol Substitution Test within 90 seconds [28]); 68.0 for women vs. 62.1 for men; *t*(94) = 2.66, *p* = .009, *d* = -0.54), whereas men demonstrated better spatial abilities than women (*n* correct on the Mental Rotation Test [29]); 11.71 for women vs. 14.48 for men; *t*(94) = -2.82, *p* = .006, *d* = 0.58).

### Stimuli, tasks, and responses

The two tasks included in both paradigms were to categorize letters as consonant or vowel and digits as odd or even using the index and middle fingers of the hand spatially corresponding to the stimulus presentation location. Stimuli appeared to left and right of a fixation cross that was presented in the middle of the screen. Stimuli presented to the left of the fixation cross were categorized with the *Y* and *X* keys of a QWERTZ keyboard and stimuli appearing to the right of the fixation cross with the *N* and *M* keys. Whereas in the letter categorization task the leftmost finger of each hand was used for consonant classification and the rightmost finger for vowel classification, the S-R mapping for the digit categorization task was counterbalanced across participants.

We employed the same stimuli, tasks, and responses as Hirsch and colleagues (2018) [11]. The stimuli consisted of a fixation cross (*+*), an asterisk, capital letters, including consonants (i.e., *G*, *K*, *M*, and *R*) and vowels (i.e., *A*, *E*, *I*, and *U*), and digits from *1* to *9* (except *5*). They appeared in white 20-pt. Arial font on a black screen. The digits and letters were presented 3 cm to the left and to the right of the fixation cross which was visible in the center of the screen throughout the entire experiment.

### Procedure

First, subjects completed a demographic questionnaire and a cognitive screening consisting of the Digit-Symbol Substitution Test [27], an *n*-back task [26], the Mental Rotation Test [29], and a matrix reasoning test [26]. Then, they performed an experiment comprising task-switching and dual-tasking (order was counterbalanced across participants). The test session including cognitive screening and experiment took about 90 min.

In both the task-switching and dual-task parts, subjects first performed one single-task block (six practice trials followed by 41 experimental trials) for each task type followed by a mixed-task/dual-task practice block (12 trials) and four mixed-task/dual-task blocks (81 trials). Finally, subjects performed another single-task block (41 trials) for each task type. Whether the first single-task block started with the digit or letter categorization task was counterbalanced across participants.

In the *task-switching part*, the stimuli were presented alternately to the left and right of the fixation cross. They disappeared after response execution and the next stimulus was presented after a random response-stimulus-interval (RSI) of 100 ms or 600 ms. In each single-task block, we presented constantly either letters or digits, so that only one task was performed in these blocks. In mixed-task blocks, we displayed both letters and digits, so that subjects repeated and switched tasks.

In single-task blocks of the *dual-task part*, a task-irrelevant asterisk was presented to the left of the fixation cross instead of Stimulus 1 and after a random SOA of 100 ms or 600 ms, a task-relevant Stimulus 2 was displayed to the right of the fixation cross. Like in the single-task blocks of the task-switching part, we presented either letters or digits as Stimulus 2, so that subjects performed only one task. In dual-task blocks, both Stimulus 1 and Stimulus 2 were task-relevant. In these blocks, letters and digits were presented as stimuli, so that there were task switches across Task 1 and Task 2 (i.e., Task 1-Task 2 switch trials) and task repetitions (i.e., Task 1-Task 2 repetition trials). The asterisk, Stimulus 1, and Stimulus 2 remained on the screen until the Task 2 response was executed. The next Stimulus 1 followed after 1,000 ms (i.e., inter-trial-interval, ITI).

The stimuli were displayed randomly with the stipulation that there were no immediate stimulus repetitions and that all stimuli were presented equally often (including practice trials). In the dual-task part, the number of task repetitions and switches across Task 2 of the previous trial and Task 1 in the current trial was almost identical (49.9% vs. 50.1%).

### Design

For the *task-switching part*, we analyzed the performance based on a 2 x 2 x 2 mixed-design with the independent between-subjects variable gender (women vs. men) and the within-subjects variables RSI (100 ms vs. 600 ms) and trial type (switch, repetition, vs. single-task). For the analysis of *mixing costs*, we contrasted repetition trials in mixed-task blocks and single-task trials, and for the analysis of *switch costs*, we compared switch trials and repetition trials in mixed-task blocks.

In the *dual-task part*, we analyzed performance in Task 1 using a 2 x 2 x 2 mixed-design with the independent between-subjects design gender (women vs. men) and the within-subjects variables SOA (100 ms vs. 600 ms) and task transition (Task 1-Task 2 switch trials vs. Task 1-Task 2 repetition trials). To assess the *PRP effect* and *switch costs* (i.e., worse performance in Task 2 in Task 1-Task 2 switch trials than Task 1-Task 2 repetition trials), we repeated the analysis for Task 2. To analyze *dual-task costs*, we employed a 2 x 2 mixed-design with the independent between-subjects variable gender (women vs. men) and the within-subjects variable task type (Task 1-Task 2 repetition trials with long SOA vs. single-task trials with long SOA). We used only Task 1-Task 2 repetition trials for the calculation of dual-task costs because these trials represent a more appropriate condition to be compared with single-task conditions. This is because in these trials Task 2 performance is unaffected by task switches, like the performance in single-task trials [11].

## Results

We discarded practice trials, the first trial in each block, and trials following an error from all data analyses. Trials with an erroneous response and trials deviating more than 3 *SD*s from each individual’s mean RT per condition (task-switching part: 1.89% of single-task trials, 2.13% of mixed-task trials; dual-task part: 1.88% of single-task trials, 1.94% of Task 1 in dual-task trials, and 1.84% of Task 2 in dual-task trials) were additionally eliminated from the RT analysis.

**Fig 1:**
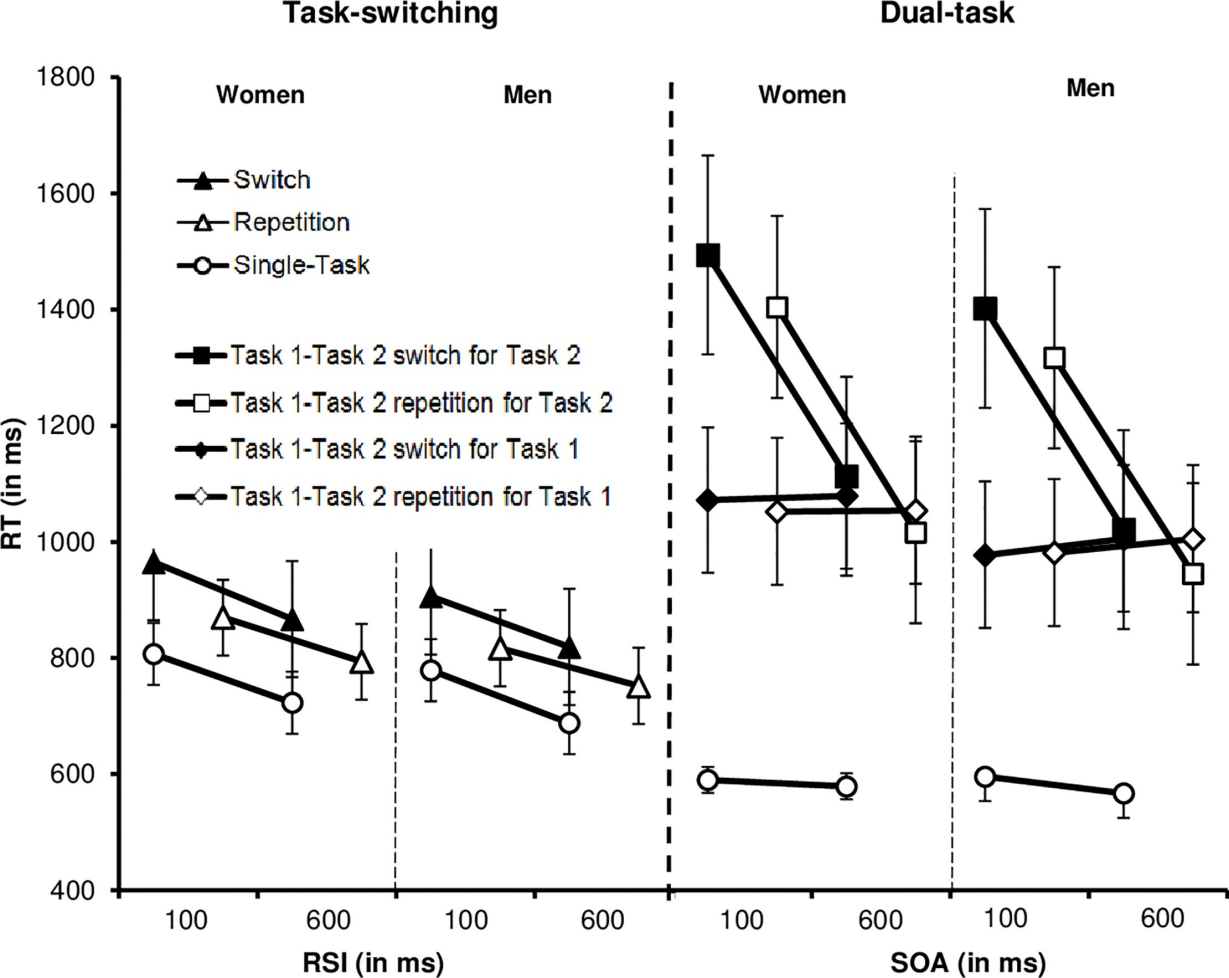
RT (ms) for task-switching as a function of trial type (switch, repetition, single-task), response-stimulus-interval (RSI, 100 ms vs. 600 ms), and gender (women vs. men) and for the dual-task for Task 1 and Task 2 as a function of task transition (Task 1-Task 2 switch trials, Task 1-Task2 repetition trials, single-task trials), stimulus onset asynchrony (SOA; 100 ms vs. 600 ms) and gender (women vs. men). Error bars represent the 95% confidence intervals.

We report the results separately for different types of performance costs in sequential (task-switching) and concurrent (dual-tasking) multitasking (Fig 1 and Table 1), followed by a covariance analysis testing the impact of the cognitive abilities showing significant gender differences (i.e., processing speed and spatial ability) on gender differences in task-switching and dual-task performance. Finally, we report Bayes statistics to determine the ratio of evidence in favor of the null hypothesis (no gender differences) vs. in favor of the alternative hypotheses (gender differences).

**Table 1:** Error rates (%; 95% confidence intervals in parenthesis) for task-switching as a function of trial type (switch, repetition, single-task), response-stimulus-interval (RSI, 100 ms vs. 600 ms), and gender (women vs. men) and for the dual-task for Task 1 and Task 2 as a function of task transition (Task 1-Task 2 switch trials, Task 1-Task 2 repetition trials, single-task trials), stimulus onset asynchrony (SOA; 100 ms vs. 600 ms), and gender (women vs. men).

	Women			Men		
**Task-switching**	**RSI 100 ms**	**RSI 600 ms**	**RSI effect**	**RSI 100 ms**	**RSI 600 ms**	**RSI effect**
Switch	4.3 (3.2–5.3)	4.3 (3.1–5.4)	-0	3.4 (2.3–4.4)	4.1 (2.9–5.3)	-0.7
Repetition	3.1 (2.3–3.9)	3.9 (3.0–4.8)	-0.8	3.1 (2.3–3.9)	3.3 (2.4–4.2)	-0.2
Single-task	3.4 (2.2–4.6)	3.3 (2.0–4.6)	-0.1	3.6 (2.5–4.8)	3.8 (2.5–5.0)	-0.2
Switch costs	-1.2	0.4		-0.3	-0.8	
Mixing costs	-0.3	0.6		-0.5	-0.5	
**Dual-task**	**SOA 100 ms**	**SOA 600 ms**	**SOA effect**	**SOA 100 ms**	**SOA 600 ms**	**SOA effect**
Task1-Task2 switch for Task1	4.0 (3.0–4.9)	7.5 (6.0–9.0)	-3.5	3.5 (2.6–4.4)	7.2 (5.7–8.7)	-3.7
Task1-Task2 repetition for Task1	4.1 (3.1–5.0)	7.3 (5.8–8.7)	-3.2	3.4 (2.5–4.3)	6.4 (5.0–7.9)	-3.0
Switch costs for Task1	0.1	0.2		0.1	0.8	
Task1-Task2 switch for Task2	7.9 (6.3–9.6)	10.1 (8.3–11.9)	-2.2	6.8 (5.2–8.4)	9.2 (7.4–11.0)	-2.4
Task1-Task2 repetition for Task2	7.6 (6.0–9.2)	8.7 (7.0–10.4)	-1.1	6.4 (4.8–8.0)	7.4 (5.6–9.1)	-1.0
Single-task	3.5 (2.3–4.7)	3.3 (1.9–4.6)	0.2	4.2 (3.0–5.4)	3.8 (2.5–5.1)	-0.4
Switch costs for Task2	0.3	1.4		0.4	1.8	
Dual-task costs	4.1	5.4		2.2	3.6	

### Task-switching performance

#### Mixing costs

For RTs, the main effects of trial type, *F*(1, 94) = 17.33, *p* < .001, η_p_^2^ = .16, and RSI, *F*(1, 94) = 173.04, *p* < .001, η_p_^2^ = .65, were significant. RTs were higher in repetition trials than in single-task trials (808 ms vs. 749 ms) and with short RSI than with long RSI (818 ms vs. 739 ms), resulting in mixing costs of 59 ms and an RSI effect of 79 ms. The interaction of trial type and RSI was significant as well, *F*(1, 94) = 5.60, *p* = .02, η_p_^2^ = .06, reflecting larger mixing costs with long than short RSI (68 ms vs. 50 ms). Critically, no gender effects reached significance (main effect of gender: *F*(1, 94) = 0.95, *p* = .33, η_p_^2^ < .01; interaction of gender and trial type: *F*(1, 94) = 0.31, *p* = .58, η_p_^2^ = .01; interaction of gender and RSI: *F*(1, 94) = 0.03, *p* = .86, η_p_^2^ < .01; interaction of gender, trial type, and RSI: *F*(1, 94) = 1.69, *p* = .20, η_p_^2^ = .02).

For the error rates, there were no significant effects (main effect of trial type: *F*(1, 94) = 0.16, *p* = .69, η_p_^2^ = .01 ; main effect of RSI: *F*(1, 94) = 2.17, *p* = .14, η_p_^2^ = .02; interaction of trial type and RSI: *F*(1, 94) = 1.98, *p* = .16, η_p_^2^ = .02), including gender effects (main effect of gender: *F*(1, 94) = 0.01, *p* = .97, η_p_^2^ < .01; interaction of gender and trial type: *F*(1, 94) = 0.73, *p* = .40, η_p_^2^ = .01; interaction of gender and RSI: *F*(1, 94) = 0.23, *p* = .63, η_p_^2^ = .01; interaction of gender, trial type, and RSI: *F*(1, 94) = 1.29, *p* = .26, η_p_^2^ = .01).

#### Switch costs

For RTs, there were significant main effects of trial type, *F*(1, 94) = 28.41, *p* < .001, η_p_^2^ = .23, and RSI, *F*(1, 94) = 107.27, *p* < .001, η_p_^2^ = .53. RTs were higher in switch trials than in repetition trials (889 ms vs. 808 ms) and with short RSI than with long RSI (890 ms vs. 808 ms), reflecting switch costs of 81 ms and an RSI effect of 82 ms. The interaction of trial type and RSI was just not significant, *F*(1, 94) = 3.96, *p* = .05, η_p_^2^ = .04, but showed a trend toward larger switch costs with short than long RSI (93 ms vs. 70 ms). Again, there were no significant gender effects, (main effect of gender: *F*(1, 94) = 0.79, *p* = .40, η_p_^2^ = .01; interaction of gender and trial type: *F*(1, 94) = 0.05, *p* = .82, η_p_^2^ = .01; interaction of gender and RSI: *F*(1, 94) = 0.48, *p* = .49, η_p_^2^ = .01; interaction of gender, trial type, and RSI: *F*(1, 94) = 0.01, *p* = .96, η_p_^2^ = < .01).

For the error rates, the main effects of trial type, *F*(1, 94) = 4.83, *p* = .03, η_p_^2^ = .05, and RSI, *F*(1, 94) = 5.20, *p* = .025, η_p_^2^ = .05, were significant. Error rates were higher in switch trials than in repetition trials (4.0% vs. 3.4%) and with long RSI than with short RSI (3.9% vs. 3.5%), reflecting switch costs of 0.6% and an RSI effect of 0.4%. The interaction of trial type and RSI, *F*(1, 94) = 0.15, *p* = .697, η_p_^2^ = .01, and, critically, all gender effects were non-significant (main effect of gender: *F*(1, 94) = 0.55, *p* = .46, η_p_^2^ = .01; interaction of gender and trial type: *F*(1, 94) = 0.14, *p* = .71, η_p_^2^ = .01; interaction of gender and RSI: *F*(1, 94) = 0.04, *p* = .84, η_p_^2^ < .01; interaction of gender, trial type, and RSI: *F*(1, 94) = 2.17, *p* = .15, η_p_^2^ = .02).

#### Summary

For task-switching performance, we found significant mixing costs and switch costs. The switch costs tended to be smaller (by 23 ms) with long RSI. In contrast, mixing costs were larger with long RSI than short RSI [8]. Importantly, there were no significant gender effects, indicating that mixing costs and switch costs were comparable across women and men.

### Dual-task performance

#### Task 1

For RTs, there were no significant effects (main effect of task transition: *F*(1, 94) = 1.42, *p* = .24, η_p_^2^ = .01; main effect of SOA: *F*(1, 94) = 1.94, *p* = .17, η_p_^2^ = .02; interaction of task transition and SOA: *F*(1, 94) = 0.12, *p* = .73, η_p_^2^ = .01). For the error rates, apart from the main effect of SOA, *F*(1, 94) = 65.32, *p* < .001, η_p_^2^ = .41, with error rates being higher with long SOA than with short SOA (7.1% vs. 3.7%), no effect was significant (main effect of task transition: *F*(1, 94) = 1.05, *p* = .31, η_p_^2^ = .01; interaction of task transition and SOA: *F*(1, 94) = 1.01, *p* = .32, η_p_^2^ = .01). Importantly, there were no gender effects in RTs (main effect of gender: *F*(1, 94) = 0.64, *p* = .43, η_p_^2^ = .01; interaction of gender and task transition: *F*(1, 94) = 2.17, *p* = .14, η_p_^2^ = .02; interaction of gender and SOA: *F*(1, 94) = 1.03, *p* = .31, η_p_^2^ = .01; interaction of gender, task transition, and SOA: *F*(1, 94) = 0.01, *p* = .98, η_p_^2^ < .01) and error rates (main effect of gender: *F*(1, 94) = 0.69, *p* = .41, η_p_^2^ = .01; interaction of gender and task transition: *F*(1, 94) = 0.66, *p* = .42, η_p_^2^ = .01; interaction of gender and SOA: *F*(1, 94) = 0.01 *p* = .98, η_p_^2^ < .01; interaction of gender, task transition, and SOA: *F*(1, 94) = 0.12, *p* = .73, η_p_^2^ = .01).

#### Task 2

For RTs, there were significant main effects of task transition, *F*(1, 94) = 55.72, *p* < .001, η_p_^2^ = .37, and SOA, *F*(1, 94) = 1255.27, *p* < .001, η_p_^2^ = .93. RTs were higher in Task 1-Task 2 switches than in repetitions (1258 ms vs. 1171 ms) and with short than with long SOA (1404 ms vs. 1024 ms), reflecting switch costs of 87 ms and a PRP effect of 380 ms. Neither the interaction of task transition and SOA, *F*(1, 94) = 0.01, *p* = .97, η_p_^2^ = .01, nor any gender effects were significant (main effect of gender: *F*(1, 94) = 0.60, *p* = .41, η_p_^2^ = .01; interaction of gender and task transition: *F*(1, 94) = 0.30, *p* = .59, η_p_^2^ = .01; interaction of gender and SOA: *F*(1, 94) = 0.12, *p* = .73, η_p_^2^ = .01; interaction of gender, task transition, and SOA: *F*(1, 94) = 0.26, *p* = .61, η_p_^2^ = .01).

For the error rates, the main effects of task transition, *F*(1, 94) = 9.89, *p* = .002, η_p_^2^ = .10, and SOA, *F*(1, 94) = 15.61, *p* < .001, η_p_^2^ = .14, were significant. Like in the RTs, there were more erroneous responses in Task 1-Task 2 switches than repetitions (8.5% vs. 7.5%), resulting in switch costs of 1.0%. Note that in contrast to the RTs, responses were more error-prone with long than short SOA (main effect of SOA; 8.8% vs. 7.2%), suggesting that the very substantial PRP effect of 380 ms was partly due to a speed-accuracy trade-off, but this was true for women and men to the same degree (see below). Moreover, the interaction of task transition and SOA was significant, *F*(1, 94) = 4.51, *p* = .036, η_p_^2^ = .05, indicating higher switch costs with long than short SOA (1.7% vs. 0.4%). Just as for the RTs, there were no significant gender effects (main effect of gender: *F*(1, 94) = 1.17, *p* = .28, η_p_^2^ = .01; interaction of gender and task transition: *F*(1, 94) = 0.2, *p* = .65, η_p_^2^ = .01; interaction of gender and SOA: *F*(1, 94) = 0.01, *p* = .93, η_p_^2^ < .01; interaction of gender, task transition, and SOA: *F*(1, 94) = 0.17, *p* = .68, η_p_^2^ = .01).

#### Dual-task costs

For RTs, there was a significant main effect of task type, *F*(1, 94) = 84.96, *p* < .001, η_p_^2^ = .48, indicating higher RTs in Task 2 of dual-task trials than in single-task trials (980 ms vs. 573 ms), resulting in dual-task costs of 407 ms. Neither the main effect of gender, *F*(1, 94) = 0.56, *p* = .46, η_p_^2^ = .01, nor the interaction of gender and task type were significant, *F*(1, 94) = 0.46, *p* = .50, η_p_^2^ = 01.

**Fig 2:**
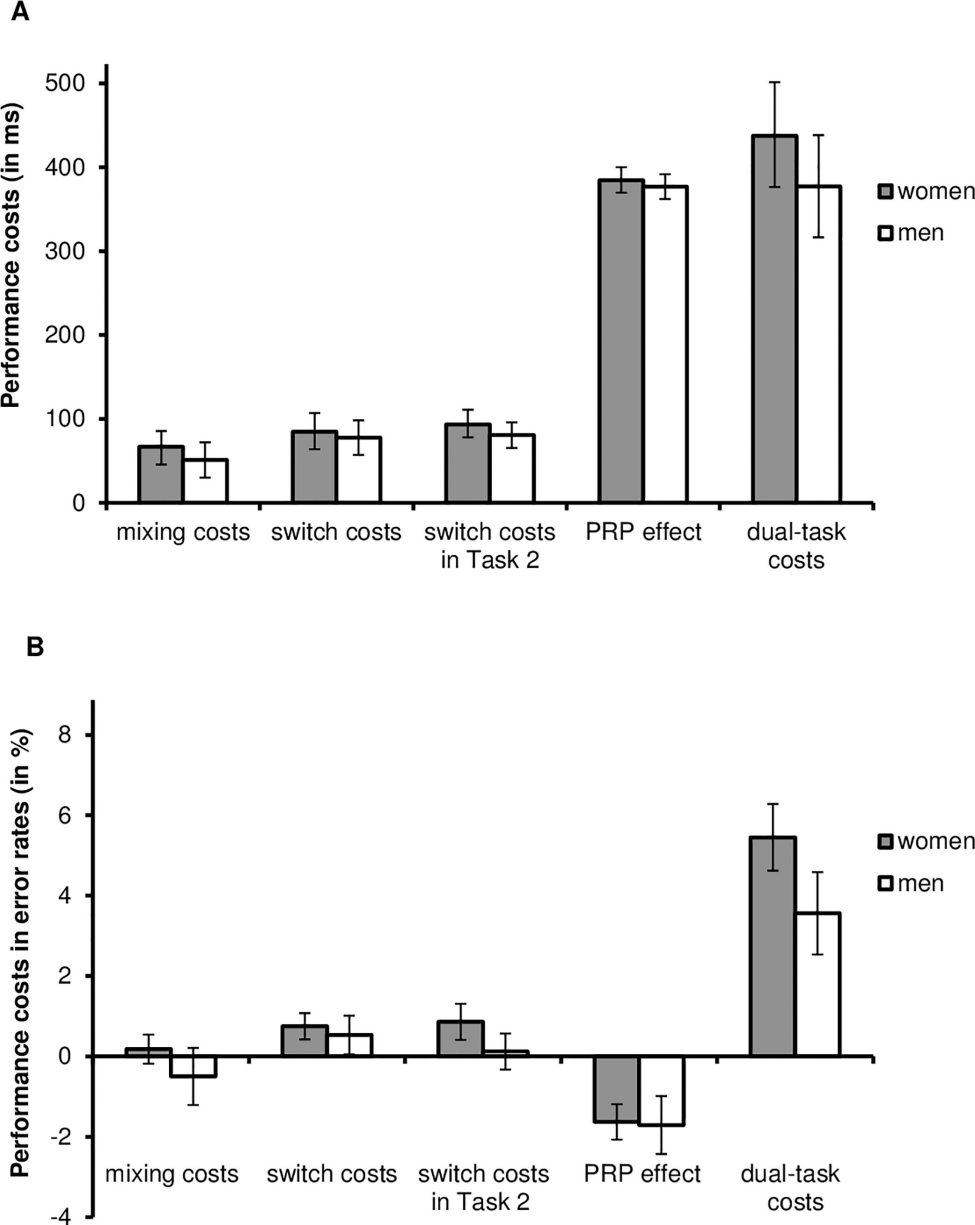
Performance costs in the reaction times (in ms, A) and in error rates (in %, B) for task-switching and dual tasking as a function of gender (women vs. men). Error bars represent the standard error.

For the error rates, the main effect of trial type was significant, *F*(1, 94) = 46.77, *p* < .001, η_p_^2^ = .33. Responses were more error prone in Task 2 of dual-task trials than in single-task trials (8.0% vs. 3.5%), resulting in dual-task costs of 4.5%. No gender effects were significant (main effect of gender: *F*(1, 94) = 0.21, *p* = .64, η_p_^2^ = .01; interaction of gender and task type: *F*(1, 94) = 2.09, *p* = .15, η_p_^2^ = .02; Fig 2).

#### Summary

For dual-task performance, we observed switch costs in Task 2 and dual-task costs. With regard to the PRP effect, there was a speed-accuracy trade-off, which, however, occurred for women and men to the same degree. Most importantly, there were no significant gender differences in any of these measures of dual-task performance.

### Additional analyses

We replicated all analyses with the processing speed and spatial ability scores as covariates (centered) to control for the significant gender differences in these abilities. The pattern of results did not change and not a single gender difference in our five multitasking measures reached significance, neither for RT nor for error rates (see Tables 2–4).

**Table 2:** Statistics for the analysis of mixing costs and switch costs in the RT and the error rates with the covariates processing speed and spatial abilities scores (centered).

	Mixing costs			Switch costs		
Effect	*F*	*p*	η_p_^2^	*F*	*p*	η_p_^2^
**RT**						
trial type	17.39	< .001	.16	28.36	< .001	.24
trial type x Digit-Symbol Substitution Test	0.25	.62	.01	1.40	.24	.02
trial type x Mental Rotation Test	2.29	.13	.02	0.07	.79	.01
trial type x gender	0.01	.96	< .01	0.19	.66	.01
RSI	176.73	< .001	.66	110.15	< .001	55
RSI x Digit-Symbol Substitution Test	0.44	.51	.01	0.06	.81	.01
RSI x Mental Rotation Test	2.48	.12	.03	3.67	.06	.04
RSI x gender	.01	.92	< .01	0.03	.87	< .01
trial type x RSI	5.52	.02	.06	3.88	.05	.04
trial type x RSI x Digit-Symbol Substitution Test	0.05	.83	.01	0.05	.83	.01
trial type x RSI x Mental Rotation Test	0.70	.41	.01	0.20	.66	.01
trial type x RSI x gender	0.77	.38	.01	0.05	.82	.01
Digit-Symbol Substitution Test	7.54	.01	.08	4.05	.05	.05
Mental Rotation Test	2.94	.09	.03	2.50	.12	.03
gender	1.39	.24	.02	0.79	38	.01
**error rates**						
trial type	0.16	.69	.01	4.91	.03	.05
trial type x Digit-Symbol Substitution Test	0.01	.97	< .01	0.31	.58	.01
trial type x Mental Rotation Test	1.12	.29	.01	3.72	.06	.04
trial type x gender	0.23	.63	.01	1.03	.31	.01
RSI	2.16	.15	.02	5.33	.02	.06
RSI x Digit-Symbol Substitution Test	0.32	.57	.01	0.02	.89	< .01
RSI x Mental Rotation Test	0.71	.40	.01	3.61	.06	.04
RSI x gender	0.12	.73	.01	0.46	.50	.01
trial type x RSI	2.04	.16	.02	0.15	.70	.01
trial type x RSI x Digit-Symbol Substitution Test	0.90	.34	.01	0.20	.66	.01
trial type x RSI x Mental Rotation Test	4.40	.04	.05	0.61	.44	.01
trial type x RSI x gender	0.04	.84	< .01	0.97	.33	.01
Digit-Symbol Substitution Test	1.73	.19	.02	2.71	.10	.03
Mental Rotation Test	0.20	.66	.01	0.05	.93	< .01
gender	0.6	.85	< .01	1.09	.30	.01

**Table 3:** Statistics for the analysis of RT and error rates in Task 1 and Task 2 with the covariates processing speed and spatial abilities scores (centered).

	Task 1			Task 2		
Effect	*F*	*p*	η_p_^2^	*F*	*p*	η_p_^2^
**RT**						
task transition	1.40	.24	.02	54.79	< .001	.37
task transition x Digit-Symbol Substitution Test	0.96	.33	.01	0.26	.61	.01
task transition x Mental Rotation Test	0.22	.64	.01	0.05	.83	.01
task transition x gender	0.87	.35	.01	0.32	.58	.01
SOA	1.96	.17	.02	1261.23	< .001	.93
SOA x Digit-Symbol Substitution Test	0.30	.58	.01	2.44	.12	.03
RSI x Mental Rotation Test	1.63	.21	.02	0.17	.68	.01
RSI x gender	1.26	.26	.01	0.70	.40	.01
task transition x SOA	0.12	.73	.01	0.01	.97	< .01
task transition x SOA x Digit-Symbol Substitution Test	0.66	.42	.01	0.96	.33	.01
task transition x SOA x Mental Rotation Test	1.27	.26	.01	1.10	.30	.01
task transition x SOA x gender	0.01	.94	< .01	0.18	.67	.01
Digit-Symbol Substitution Test	7.09	.01	.07	10.12	.01	.10
Mental Rotation Test	0.06	.82	.01	< .01	.99	< .01
gender	2.25	.14	.02	2.44	.12	.03
**error rates**						
task transition	1.06	.31	.01	9.84	.01	.10
task transition x Digit-Symbol Substitution Test	1.65	.20	.02	1.45	.23	.02
task transition x Mental Rotation Test	0.50	.48	.01	0.38	.54	.01
task transition x gender	0.35	.56	.01	0.79	.38	.01
SOA	68.60	< .001	.43	15.32	< .001	.14
SOA x Digit-Symbol Substitution Test	2.81	.10	.03	0.06	.81	.01
RSI x Mental Rotation Test	1.83	.18	.02	0.25	.62	.01
RSI x gender	0.01	.97	< .01	0.02	.90	< .01
task transition x SOA	1.03	.31	.01	4.43	.04	.05
task transition x SOA x Digit-Symbol Substitution Test	4.02	.04	.04	0.25	.62	.01
task transition x SOA x Mental Rotation Test	0.04	.83	< .01	0.01	.98	< .01
task transition x SOA x gender	0.07	.79	.01	0.06	.81	.01
Digit-Symbol Substitution Test	2.89	.09	.03	0.07	.80	.01
Mental Rotation Test	0.85	.36	.01	6.74	.01	.07
gender	0.01	.96	< .01	0.13	.72	.01

**Table 4:** Statistics for the analysis of dual-task costs in RT and error rates with the covariates processing speed and spatial abilities scores (centered).

Effect	*F*	*p*	η_p_^2^
**RT**			
task type	887.33	< .001	.91
task type x Digit-Symbol Substitution Test	3.88	.05	.04
task type x Mental Rotation Test	0.13	.72	.01
task type x gender	0.92	.32	.01
Digit-Symbol Substitution Test	11.07	.01	.11
Mental Rotation Test	0.01	.98	< .01
gender	2.55	.11	.03
**error rates**			
task type	4.33	.04	.05
task type x Digit-Symbol Substitution Test	0.01	.94	< .01
task type x Mental Rotation Test	0.19	.66	.01
task type x gender	0.06	.81	.01
Digit-Symbol Substitution Test	0.40	.53	.01
Mental Rotation Test	5.48	.02	.06
gender	0.38	.54	.01

Moreover, we repeated all the analyses with the additional between-subjects independent variable testing location (i.e., Aachen vs. Frankfurt). All effects including the between-subjects variables gender and testing location and the within-subjects variables task transition or task type were not significant, meaning that there were no gender effects in multitasking costs at both testing locations, all *F*s < 1.39 and all *p*s > . 24 in RTs, and all *F*s < 1.51 and all *p*s > .22 in the error rates.

Since our analyses showed a lack of evidence for gender differences in task-switching and dual-task performance, we additionally computed Bayes factors [30, 31]. Bayes factors can be used to interpret results that did not reach an alpha of .05 because they quantify the support for the null hypothesis over the alternative hypothesis and thus provide information about the strength of evidence for the lack of gender differences in multitasking performance. We employed the method proposed by Rouder and colleagues (2009) [32] and analyzed scaled JZS Bayes factors (scale *r* = 1) for the critical interactions including the between-subjects variable gender.

The Bayes factors (for the data set including subjects at both testing locations) showed evidence in favor of the null hypothesis for all performance costs, including mixing costs (RT: Bayes factor _0A_ = 5.528; error rates: Bayes factor _0A_ = 4.539), switch costs in task-switching (RT: Bayes factor _0A_ = 6.228; error rates: Bayes factor _0A_ = 5.979), switch costs in Task 2 of the dual-task (RT: Bayes factor _0A_ = 5.545; error rates: Bayes factor _0A_ = 5.799), the PRP effect (RT: Bayes factor _0A_ = 6.017; error rates: Bayes factor _0A_ = 6.357), and dual-task costs (RT: Bayes factor _0A_ = 5.138; error rates: Bayes factor _0A_ = 2.410). Bayes factors between three and ten have been proposed to indicate substantial evidence and Bayes factors between one and three have been suggested to indicate anecdotal evidence [33]. Note that the Bayes factors in the present study provide no decisive evidence (Bayes factors > 100) against the existence of gender differences in multitasking (i.e., null hypothesis) but they consistently speak against gender differences in all of these performance costs. Put differently, we found no discernible evidence in favor of gender differences in multitasking using a study in which we tested multiple indices of multitasking in commonly used experimental paradigms (task switching and dual tasks), even though we have good statistical power (with N = 96) to detect such effects of at least medium-to-large size, if they were present.

## Discussion

The general aim of the present study was to systematically explore gender performance differences in a range of measures of multitasking costs occurring in task-switching and dual-task situations while controlling for gender differences in relevant cognitive abilities. To this end, participants performed highly comparable task-switching and dual-task paradigms and a cognitive test battery.

Consistent with previous studies, we observed substantial multitasking costs including mixing costs and switch costs in the task-switching paradigm, and the PRP effect, switch costs in Task 2, and dual-task costs in the dual-task paradigm [9, 11, 34]. Note that in contrast to previous studies, mixing costs were larger with long than short RSI and that there was a slight speed-accuracy trade-off for the PRP effect. However, the increased mixing costs with long RSI and the trade-off occurred for both women and men to a comparable extent and is, therefore, potentially relevant only when interpreting these multitasking effects per se; however they do not affect our general conclusions about gender-specific differences in multitasking performance.

Most importantly, none of the observed multitasking costs differed in size across gender, indicating that women and men performed equally well in both sequential and concurrent multitasking situations. Even when controlling for processing speed and spatial abilities, which, in line with prior studies, differed across gender groups (see, e.g., [35] for a review on gender differences in processing speed, and [36] for a meta-analysis on gender differences in spatial abilities), differences in multitasking costs across women and men remained absent.

The absence of any gender difference in task-switching and dual-task performance is not in line with the findings of Stoet and colleagues (2013) [17] and by Mäntylä (2013) [19] who observed better multitasking performance for women than for men, or vice versa. A major difference between the present study and the task-switching study by Stoet and colleagues (2013) [17] lies in “stimulus valence”. In order to focus on the divided attention and attention shifting component of multitasking, we used univalent stimuli in the present study. Whereas bivalent stimuli activate both task sets (and hence induce substantial interference on the stimulus level), the univalent stimuli used in our study were only associated with one task (i.e., letters do not afford the digit categorization task and vice versa) (see [8] for a review) and require less selective attention because the relevant stimulus attribute is cued by the spatial location of the stimulus presentation. Thus, based on our data, we cannot completely exclude gender effects in specific aspects of selective attention when processing bivalent stimuli.

Concerning dual-task performance, Mäntylä (2013) [19] observed that men outperformed women. However, he used a specific dual-task situation with three independent monitoring tasks and a working memory task. In contrast, we employed a typical dual-task paradigm and used discrete RT tasks (i.e., tasks with a definitive start and end point) instead of continuous tasks (i.e., tasks which are performed over several minutes and in which the number of correctly solved tasks is analyzed [37]). Since there are some studies that did not find gender effects with continuous tasks [10], the task type (i.e., discrete vs. continuous) seems to have no obvious impact on the occurrence of gender effects in dual-tasking. Rather, the seemingly contradictory findings in the present study and the study by Mäntylä (2013) [19] may be attributable to different dual-task paradigms and the cognitive demands posed by these paradigms. For example, in contrast to the present study, the study by Mäntylä (2013) involves offloading to spatial representations, which may be related to gender differences in spatial abilities.

Hence, small gender differences in multitasking abilities across women and men in the used task-switching and dual-task paradigms cannot be excluded based on the present study. Moreover, the present study does not allow any conclusions about gender differences in other multitasking situations, which for example call for more planned and future-oriented strategies or involve offloading of spatial abilities [19]. However, considering the good power of the present study to detect even medium-to-large gender differences, the present findings strongly suggest that there are no substantial gender differences in multitasking performance across task-switching and dual-task paradigms, which predominantly measure cognitive control mechanisms such as working memory updating, the engagement and disengagement of task sets, and inhibition.
